# Mapping Light-Dressed Floquet Bands by Highly Nonlinear Optical Excitations and Valley Polarization

**DOI:** 10.1021/acs.jpclett.3c02936

**Published:** 2023-12-08

**Authors:** Anna Galler, Angel Rubio, Ofer Neufeld

**Affiliations:** † Max Planck Institute for the Structure and Dynamics of Matter, Center for Free Electron Laser Science, 22761 Hamburg, Germany; ‡ Center for Computational Quantum Physics, Flatiron Institute, New York, New York 10010, United States

## Abstract

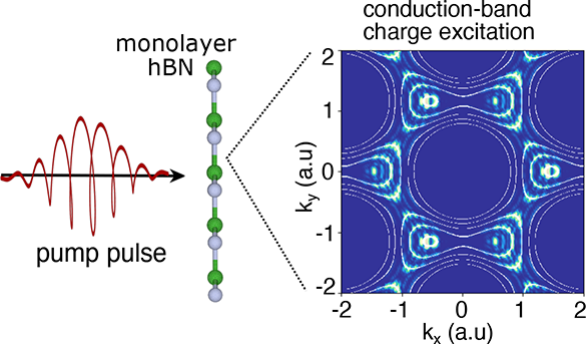

Ultrafast nonlinear optical phenomena in solids have been attracting a great deal of interest as novel methodologies for the femtosecond spectroscopy of electron dynamics and control of the properties of materials. Here, we theoretically investigate strong-field nonlinear optical transitions in a prototypical two-dimensional material, hBN, and show that the *k*-resolved conduction band charge occupation patterns induced by an elliptically polarized laser can be understood in a multiphoton resonant picture, but, remarkably, only if using the Floquet light-dressed states instead of the undressed matter states. Our work demonstrates that Floquet dressing affects ultrafast charge dynamics and photoexcitation even from a single pump pulse and establishes a direct measurable signature for band dressing in nonlinear optical processes in solids, opening new paths for ultrafast spectroscopy and valley manipulation.

In recent years, strong-field physics and nonlinear optical processes in solids have been intensely investigated.^1−6^ Such processes allow the probing and manipulating of ultrafast electron dynamics and properties of materials with potential attosecond resolution.^7−9^ For instance, high-harmonic generation (HHG) provides routes for exploring dynamical correlations,^10−13^ electron–phonon coupling,^14,15^ spectral caustics,^16^ exciton formation and dissociation,^17^ topology,^18−22^ and more.^5,23,24^ Nonlinear photocurrent generation similarly allows investigation of electron coherence and correlations.^22,25−27^ Specifically in the realm of two-dimensional (2D) hexagonal materials with valley degrees of freedom,^28−30^ intense femtosecond lasers have been used to nonresonantly control and read the valley pseudospin,^25,31−38^ which has technological implications for petahertz electronics, spintronics, and memory devices.

In all of these examples, electronic transitions from the valence to the conduction bands play a pivotal role, e.g., by determining the valley polarization or by forming the essential first step in HHG and the photogalvanic effect. Notably, the transition amplitude is determined by the nature of the involved electronic states. Moreover, the specific shape of the bands affects the real-time dynamics of propagation of an electron through the material. Consequently, it is crucial to ascertain which electronic states are involved: the field-free ones or the light-dressed ones? The answer to this question is essential not only for our fundamental physical understanding but also for formulating all-optical electronic-structure reconstruction techniques^39−42^ and for Floquet material engineering.^43−45^ Nonetheless, only a few works to date have considered possible modifications of the electronic bands of solids in strong-field processes,^23,33,46,47^ and none in the context of multiphoton transitions from the driving field itself (i.e., without a secondary probe pulse “sensing” the dressed states, but where the pulse both dresses the solid and senses the dressing it itself created). While ref (48) analyzed transitions between instantaneous Floquet states in a two-band model, the overwhelming assumption in most works is that the field-free bands are a good basis for interpreting the strong-field dynamics. In this context, previous works also reported a plethora of unique charge excitation patterns upon strong-field driving, which were suspected to arise from multichannel interference,^25,31,33,35^ but their exact microscopic origin remained unexplained.

Here we theoretically investigate strong-field light-driven excitations in a prototypical 2D material using both sophisticated lattice models and ab initio time-dependent density functional theory (TDDFT). We explore the direct connection between the electronic structure and the induced conduction band (CB) charge excitation patterns driven by intense elliptically polarized lasers. We find that the resulting CB occupations do not respect the symmetries of the field-free bands or uphold the naively expected energy conservation at multiphoton transitions between field-free states. This unambiguously indicates that laser dressing plays a major role in the dynamics, even with a single pump field. Interestingly, the CB occupations do follow a clear multiphoton resonant picture if the Floquet light-dressed states are employed instead of the field-free ones. We investigated how this result affects nonlinear valley selectivity. Our work establishes evidence for light-induced electronic structure in nonlinear optics that should be directly detectable with time- and angle-resolved photoelectron spectroscopy (tr-ARPES)^45,49,50^ and has significant implications for other nonlinear phenomena.

We start by analyzing laser-induced CB charge excitation patterns in a generic 2D model system that exhibits valley degrees of freedom. To this end, we employ a real-space 2D model for a general honeycomb lattice with dissimilar A/B sublattice sites and periodic boundary conditions, where each site is formed from a local Gaussian potential well (for details of the lattice model, see the Supporting Information). The result is a honeycomb lattice with broken inversion symmetry with two electrons per unit cell, leading to a spectrum with a direct optical gap at the *K*/*K*′ points (see Figure 1). Note that each Gaussian locally supports an s-like atomic state, but the hybridization leads to non-zero electronic angular momenta and Berry curvature in both of the *K*/*K*′ valleys. Thus, this is the simplest possible real-space model for a system with valley degrees of freedom, in which only one dominant valence and conduction bands play a role. For our chosen parameters (an 8% difference in the potentials of the A/B sites), we obtain a direct optical gap of ∼2 eV at *K* and *K*′ (see Figure 1c). We simulate the interaction of this electronic system with an intense elliptically polarized laser pulse (up to ∼0.2 TW cm^–2^), with a nonresonant carrier frequency that is well below the band gap. The numerical methodology consists of solving the time-dependent Schrödinger equation for the electronic dynamics (employing the dipole approximation), while assuming the independent particle approximation (i.e., neglecting electron–electron interactions). All technical details of the simulations are available in the Supporting Information. Importantly, due to the nonresonant conditions, highly nonlinear optical processes involving multiple photons are required to excite electrons from the valence to the conduction bands (see the illustration in Figure 1c). The resulting charge distribution patterns in the CB after the laser pulse ends are calculated by projecting the final states onto the field-free states. Figure 2 presents such exemplary spectra for several driving conditions, showing the emergence of distinct ringlike patterns.

**Figure 1:**
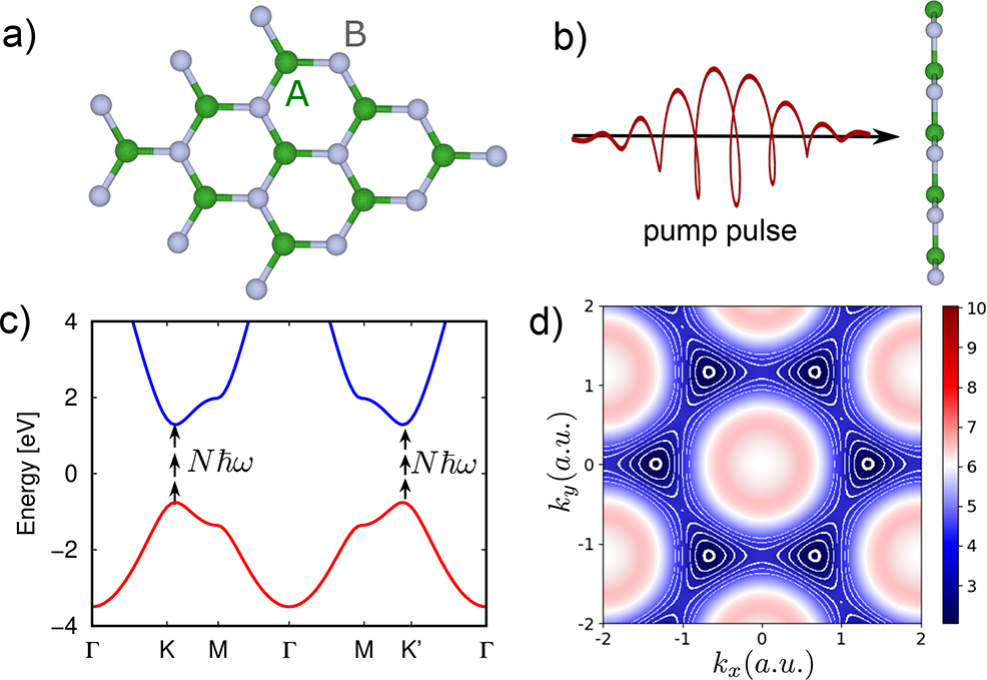
(a) Honeycomb lattice (top view) with inequivalent A/B sublattice sites. (b) An elliptically polarized intense pump pulse excites electrons from the valence to the conduction bands. The frequency of the laser pulse is well below the system’s band gap, requiring multiphoton transitions (illustrated in panel c). (c) Equilibrium band structure of the employed model along high-symmetry lines (red and blue denote occupied and unoccupied states, respectively). (d) Equilibrium *k*-resolved band gap of the employed model. The white lines indicate direct gap energies resonant with multiples of the driving photon energy (multiphoton resonant contours).

**Figure 2:**
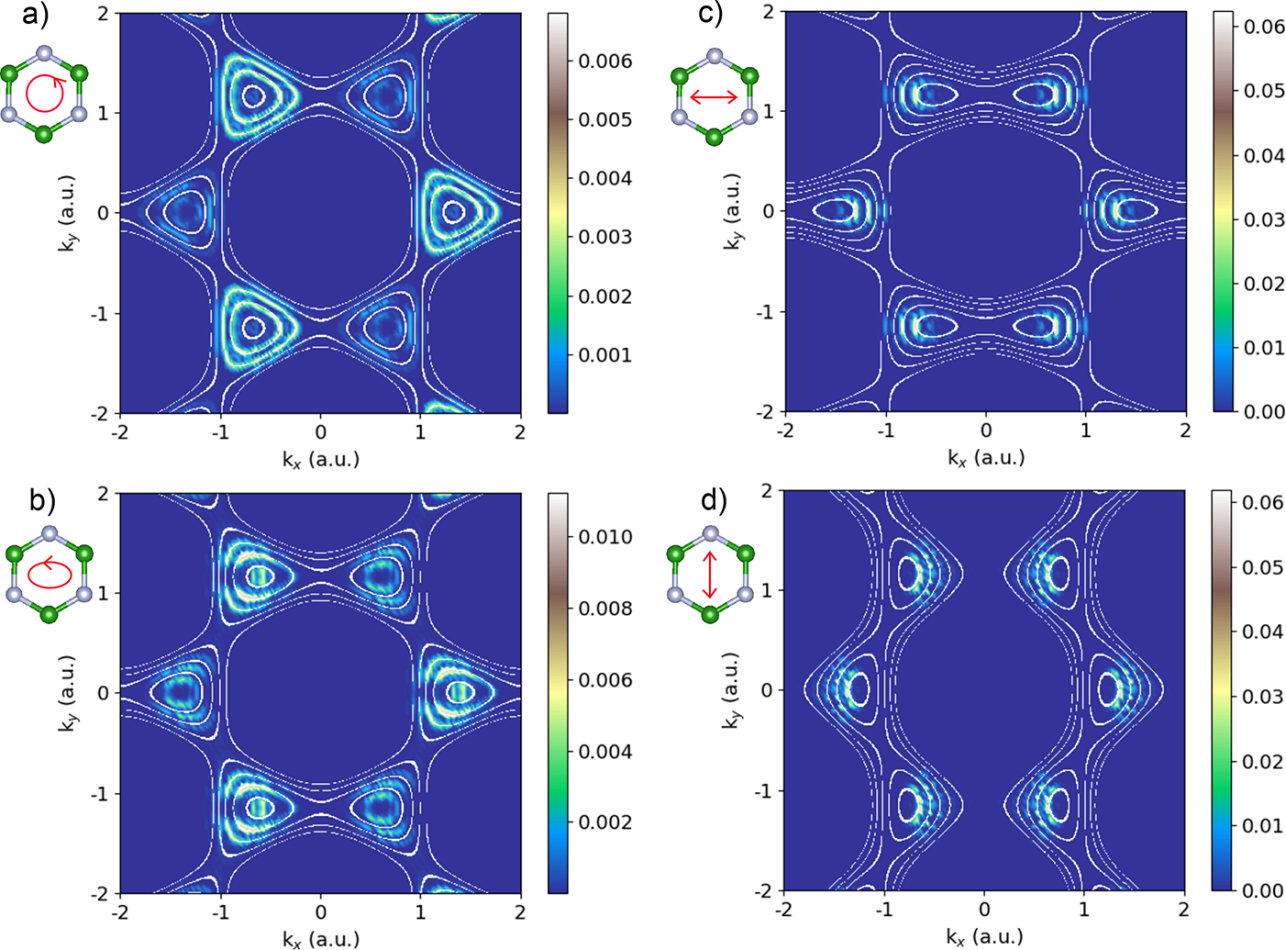
Conduction band charge excitation patterns in the model. (a) For a circularly polarized pump pulse, with a photon frequency of 0.35 eV and a laser intensity of 0.2 TW/cm^2^. The white lines indicate energies of the Floquet *k*-resolved direct gap resonant with integer multiples of the driving frequency. (b) Same as panel a, but for an elliptically polarized driving pulse with an ε of 0.6. (c) Charge excitation pattern for linear driving in the *x*-direction. (d) Under linear driving in the *y*-direction.

Let us first analyze the induced occupations under circular driving (Figure 2a). Here the ring-shaped patterns form a triangular shape reminiscent of the trigonal wrapping of the Brillouin zone (BZ) in the field-free bands of the honeycomb system, e.g., as reflected in the *k*-dependent gap in Figure 1d. Thus, our initial suspicion is that the shape of the ground-state bands is imprinted onto the *k*-space occupation patterns induced by an external light field. Before this hypothesis is explored, it is important to note that, within the dipole approximation and our employed methodology, only direct optical transitions that conserve the electron crystal momentum are considered. Thus, one expects that the transition amplitude and final CB occupations *g*_CB_ would be determined by a Fermi’s golden rule expansion of the form (to first order in perturbation theory): *g*_CB_^(1)^(**k**, *t*) = −*i*∫_0_^*t*^ d*t*⟨ψ_**k**,v_|*V*_int_(*t*)|ψ_**k**,c_⟩e^*iΔε*_CV_(**k**)*t*^, where *V*_int_ is the laser–matter interaction, |ψ_**k,**v/c_⟩ represents the field-free Bloch state at **k**, *t* would eventually be taken at the end of the pulse *t* = *t*_f_, and *Δε*_CV_(**k**) = ε_c_(**k**) – ε_v_(**k**) is the energy separation between the bands at **k**. Generally, higher orders of perturbation theory are required for multiphoton processes, with the following *n*th-order term:
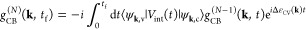
1From eq 1, the amplitude of *N*-photon absorption is proportional to *E*_0_^*N*^ (as expected for a perturbative theory). The CB occupations can be expressed as a sum of multiphoton processes: *g*_CB_(**k**) = |∑_*n*=1_^∞^*g*_CB_^(*N*)^(**k**, *t*_f_)|^2^. Importantly though, because the laser is monochromatic, the integral “selects” energies that are resonant with a multiphoton condition, i.e., *k*-points that uphold *Δε*_CV_(**k**) = *nω*, where *n* is any integer (equivalent to energy conservation). Consequently, for every given *k* point, only one term in the series would dominantly contribute (the term for which the multiphoton transition is closest to the *k*-dependent gap).

If we overlay the resonant multiphoton lines obtained from the ground-state system (Figure 1d) onto the resulting occupation patterns driven by circularly polarized light (Figure 2a), they do not match; only the expected qualitative shape of the triangular wrapping is reproduced, while energies are shifted in some conditions by a maximal displacement of ∼0.5ω (see the Supporting Information). The situation worsens if we consider other laser polarizations, where trigonal wrapping is lost (Figure 2b–d). Overall, it is obvious that this multiphoton picture is too simplistic. It is also clear that the symmetries of the laser–matter system play a role in determining the final electronic occupation patterns. One might wonder if this picture fails due to its perturbative nature (describing a not necessarily perturbative process). However, we recall that only the transition amplitudes would change in the nonperturbative approach, but the transition energies should still fulfill the energy conservation condition that holds regardless of being in the perturbative or nonperturbative regimes.

At this stage, we consider an alternative explanation for the CB occupation patterns that relies on a laser-dressing picture for the electronic system. From a formal perspective, the coupling of the electronic system to the laser in the model is performed with a Peierls substitution.^51,52^ One expects the electron momenta to couple to the laser vector potential **A**(*t*), and **k** → **k**(*t*) = **k** – **A**(*t*)/*c*. Consequently, the field-free band structure can be considered to be effectively “shaking” in time along the laser polarization axis, opening up other resonant multiphoton channels between different regions in *k*-space. The resulting transitions should reflect a complicated time average of those available channels, weighted by the particular intensity of the laser and the density of states in each moment in time. Such a picture allows rotational symmetry breaking and should shift the peaks from the ground-state resonance conditions, as observed numerically. Mathematically, however, it is not clear how the averaging procedure should be performed.

An alternative but equivalent description can be achieved with Floquet theory, where the “shaken” bands are replaced by the quasi-energy bands.^53^ The quasi-energy bands are eigenstates of the quantum propagator and have constant occupations, allowing non-ambiguous description of the dynamics. This is the standard approach for describing laser-dressing effects in many time-periodic systems (see, e.g., refs (54−56)) and describes phenomena from HHG^57,58^ to dynamical Franz Keldysh effects.^59−61^ Thus, a natural extension of the hypothesis presented above is to simply replace the ground-state Bloch states in Fermi’s golden rule with Floquet–Bloch states, generating a resonant condition at *k*-points that uphold ε_c_^F^(**k**) – ε_v_^F^(**k**) = *nω*, where ε_c_^F^(**k**) and ε_v_^F^(**k**) are the Floquet quasi-energy bands, which are light-dressed and differ from ε_c_(**k**) and ε_v_(**k**). Let us emphasize that it is *a priori* not clear whether such a replacement is legitimate. Even if the Floquet states are eigenstates of the driven system, it is not obvious that one could formulate a Fermi’s golden rule with them, because (i) the states are time-dependent, (ii) the interaction with the laser is already incorporated into the states themselves, (iii) there are ambiguities in determining the ordering and precise eigenenergies of the states, and (iv) Fermi’s golden rule arises from time-dependent perturbation theory, whereas the Floquet states are nonperturbative entities.

To test this hypothesis, we construct a two-band tight-binding (TB) Hamiltonian *H*(**k**) for the model, including up to fifth-order nearest-neighbor (NN) hopping terms (following ref (45); see also ref (62)). All hopping terms were fitted such that *H*(**k**) reproduces the bands obtained from the real-space model (see the Supporting Information for details). The TB Hamiltonian is expected to correctly capture the generic electron dynamics induced by the laser field, while deviations are expected due to the Hamiltonian not including the full real-space dynamics of Bloch states. We couple *H*(**k**) to an external laser through a Peierls substitution and calculate the corresponding Floquet quasi-energy bands of the light-driven Hamiltonian assuming perfect temporal periodicity (neglecting the laser envelope^63^). The resulting time-dependent Hamiltonian *H*(**k**(*t*)) is decomposed into harmonics of ω to obtain the sub-blocks of the Floquet Hamiltonian:
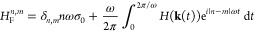
2where |*n* – *m*| is the photon channel order and the integrals are numerically solved for each *k*-point. Subsequently, the Floquet Hamiltonian is diagonalized to obtain quasi-energies, which are corrected by their photon-channel index. The resulting light-dressed bands, ε_c_^F^(**k**) and ε_v_^F^(**k**), are taken as the bands that converge to the correct field-free bands in the limit of zero laser power.

Figure 2 presents the main result of this Letter; it compares the numerically obtained charge excitation patterns (i.e., from directly solving the time-dependent Schrödinger equation for the real-space model coupled to laser driving) with multiphoton resonant energy contours in the light-dressed bands (overlaid in white). The two match very well in all of the examined laser regimes, including different laser wavelengths, powers, and polarizations (see the Supporting Information). Thus, the numerical results validate the hypothesis presented above. Notably, there is an intuitive connection between eq 1 employed with Floquet–Bloch states (instead of the field-free Bloch states) to Floquet physics that is worth mentioning. Because each Floquet–Bloch state’s quasi-energy is determined modulo an integer number of photon energies, the interaction term does not practically alter the energy conservation condition when acting on the Floquet–Bloch states. In that respect, the hypothesized Fermi golden expression can be replaced with a nonperturbative description that evaluates the overall overlap between valence- and conduction-associated Floquet–Bloch states:
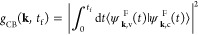
3This allows interpretation of band occupations after the pulse as arising from the time average of overlaps of the nonperturbative Floquet–Bloch states associated with the dressed bands.

We further discuss some noteworthy points. (i) The exceptionally good matching between the CB occupations and the resonant multiphoton transition picture in the light-dressed system means that the driving laser both dresses the system and induces the optical transitions. Consequently, our results propose an observable that is directly sensitive to light dressing without an additional probe pulse. (ii) In the case of a circularly polarized laser driving (Figure 2a), the triangular-shaped rings around *K*/*K*′ reflect the shape of the Floquet bands; because the circular pulse respects the 3-fold rotational symmetry of the lattice,^64,65^ trigonal wrapping is preserved. (iii) The *K*/*K*′ valleys couple differently to circularly polarized components of the laser due to the valley degrees of freedom, and the direct gaps at *K* and *K*′ differ. Crucially, the latter is the origin of the different charge excitation patterns in the *K* and *K*′ valleys in this nonresonant intense driving regime. Together with optical selection rules based on the orbital angular momentum in the valleys,^66,67^ this effect gives rise to valley asymmetry in the highly nonlinear nonresonant regime. It is also noteworthy that in the high-frequency and/or weak-driving limits, the Floquet bands coincide with the field-free bands (see the Supporting Information), and the standard Fermi golden rule picture is restored. In that respect, to observe any interesting occupation patterns, there need to be strong band modifications in the first place. (iv) For generic elliptical driving, the CB occupations become compressed along or transverse to the driving axis. This provides an all-optical knob for tuning the valley selectivity. (v) We note some discrepancies in the agreement between the numerical results and the Floquet multiphoton contours at *K*′ (but not at *K*). We have verified that this discrepancy is not a result of the laser parameters and likely arises from inaccuracies in the tight-binding model itself accompanied by multiband Floquet transitions (see the Supporting Information for further discussion). (vi) In very intense driving regimes, this simple picture breaks down due to transitions involving multiple Floquet bands (which could also arise in other driving regimes^48^). Nevertheless, in the Supporting Information we show that the structure of the occupation patterns still follows the structure of the Floquet bands, suggesting that the picture remains indicative of the induced electron dynamics.

Next, we validate this model result in a realistic 2D material. We perform ab initio calculations for a monolayer of hexagonal boron nitride (hBN) irradiated by an intense laser pulse with a frequency of 0.7 eV, well below the band gap of hBN (4.2 eV with the local density approximation). We employ a real-time TDDFT approach as implemented in the Octopus^68−71^ code. The methodology is similar to that employed for the model presented above but with multiple optically active valence electrons that interact with each other as well as with the driving laser (for details, see the Supporting Information). Figure 3a–d shows the corresponding CB excitation patterns for several driving conditions. The patterns are overlaid with the multiphoton resonant transition contours obtained for the Floquet quasi-energy bands from a TB model with the TB parameters fitted to the hBN first valence and conduction bands. Overall, the induced patterns agree remarkably well with the Floquet resonant transitions and effectively map the light-dressed bands. Small deviations can be observed here because the TDDFT calculations include excitations from multiple valence bands to multiple conduction bands as well as electron–electron interactions, both of which are neglected in the Floquet TB approach.

**Figure 3:**
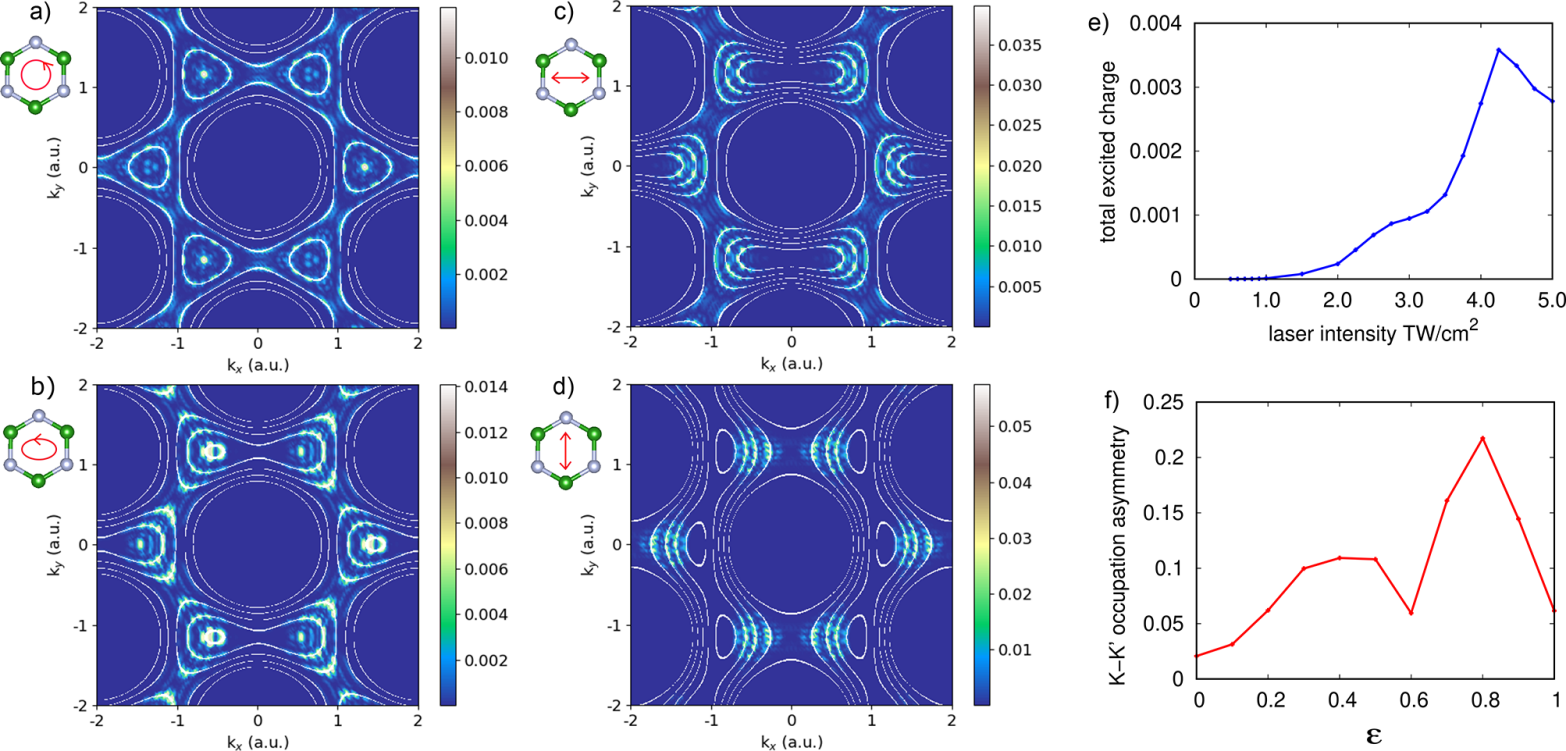
Charge excitation patterns and highly nonlinear valley asymmetry in hBN. (a) Conduction band charge excitation pattern under circular driving, (b) an elliptically polarized pump pulse with an ε of 0.6, (c) a linearly polarized pulse in the *x*-direction, and (d) linear driving in the *y*-direction. In all depicted cases, the laser intensity is 2 TW/cm^2^ and the photon frequency is 0.7 eV. The white lines indicate energies of the Floquet *k*-resolved direct gap resonant with integer multiples of the driving frequency. (e) Total excited charge in the conduction bands vs laser intensity. (b) Valley polarization in hBN vs driving ellipticity (for a laser power of 2 TW/cm^2^).

Figure 3f further shows a quantitative measure for the valley asymmetry in hBN, defined as *P* = (*n*_*K*_ – *n*_*K*′_)/(*n*_*K*_ + *n*_*K*′_), where *n*_*K*_ (*n*_*K*′_) is the electron occupation in each valley obtained by integrating the population around *K* (*K*′). The valley polarization increases with driving ellipticity ε. However, the increase is not monotonic, unlike in the resonant case.^66^ We believe that this is a testament to the complex CB occupation patterns obtained in the highly nonlinear regime. Indeed, Figure 3e shows that the total CB excitation under these conditions is a nonperturbative nonlinear observable. Thus, our results could form a new approach for analyzing and predicting valley asymmetry under strong-field and nonresonant driving.

To summarize, in this work we investigated strong-field optical excitations in 2D materials. We found that the excitations can be analyzed within a multiphoton resonant picture, but only if the Floquet light-dressed states are employed. Importantly, the *k*-space distribution of electrons in the conduction and valence band after the laser pulse effectively maps the light-driven Floquet bands, including symmetry breaking induced by the laser. These phenomena can be directly experimentally accessed by ARPES performed after the laser pulse ends (i.e., indirectly, where the ARPES probe does not temporally overlap with the Floquet-dressed phase).^27,45,49,50,72^ While we explored here 2D hexagonal materials, the results should be general with respect to other periodic systems. Since our findings establish a clear connection between the light-dressed electronic structure and the material’s nonlinear optical excitations, they should affect research in all connected fields such as HHG, harmonic side band generation, and nonlinear photogalvanic effects, implying possible new interpretations of ultrafast spectroscopies based on these techniques.

## Supporting Information Available

Technical details concerning the model and TDDFT calculations and additional results for several field strengths, frequencies, and pulse lengths (PDF)

## References

[ref1] Ghimire S.; Ndabashimiye G.; DiChiara A. D.; Sistrunk E.; Stockman M. I.; Agostini P.; DiMauro L. F.; Reis D. A. Strong-field and attosecond physics in solids. Journal of Physics B: Atomic, Molecular and Optical Physics 2014, 47, 204030 10.1088/0953-4075/47/20/204030.

[ref2] Ghimire S.; Reis D. A. High-harmonic generation from solids. Nat. Phys. 2019, 15, 10–16. 10.1038/s41567-018-0315-5.

[ref3] Sederberg S.; Zimin D.; Keiber S.; Siegrist F.; Wismer M. S.; Yakovlev V. S.; Floss I.; Lemell C.; Burgdörfer J.; Schultze M.; et al. Attosecond optoelectronic field measurement in solids. Nat. Commun. 2020, 11, 430 10.1038/s41467-019-14268-x. 31969568 PMC6976600

[ref4] Sederberg S.; Kong F.; Hufnagel F.; Zhang C.; Karimi E.; Corkum P. B. Vectorized optoelectronic control and metrology in a semiconductor. Nat. Photonics 2020, 14, 680–685. 10.1038/s41566-020-0690-1.

[ref5] Yue L.; Gaarde M. B. Introduction to theory of high-harmonic generation in solids: tutorial. J. Opt. Soc. Am. B 2022, 39, 535–555. 10.1364/JOSAB.448602.

[ref6] Park J.; Subramani A.; Kim S.; Ciappina M. F. Recent trends in high-order harmonic generation in solids. Adv. Phys.: X 2022, 7, 2003244 10.1080/23746149.2021.2003244.

[ref7] Langer F.; Hohenleutner M.; Schmid C. P.; Pöllmann C.; Nagler P.; Korn T.; Schüller C.; Sherwin M.; Huttner U.; Steiner J.; et al. Lightwave-driven quasiparticle collisions on a subcycle timescale. Nature 2016, 533, 225–229. 10.1038/nature17958. 27172045 PMC5034899

[ref8] Baudisch M.; Marini A.; Cox J. D.; Zhu T.; Silva F.; Teichmann S.; Massicotte M.; Koppens F.; Levitov L. S.; García de Abajo F. J.; et al. Ultrafast nonlinear optical response of Dirac fermions in graphene. Nat. Commun. 2018, 9, 1018 10.1038/s41467-018-03413-7. 29523791 PMC5844892

[ref9] Reimann J.; Schlauderer S.; Schmid C.; Langer F.; Baierl S.; Kokh K.; Tereshchenko O.; Kimura A.; Lange C.; Güdde J.; et al. Subcycle observation of lightwave-driven Dirac currents in a topological surface band. Nature 2018, 562, 396–400. 10.1038/s41586-018-0544-x. 30258232

[ref10] Silva R.; Blinov I. V.; Rubtsov A. N.; Smirnova O.; Ivanov M. High-harmonic spectroscopy of ultrafast many-body dynamics in strongly correlated systems. Nat. Photonics 2018, 12, 266–270. 10.1038/s41566-018-0129-0.

[ref11] Tancogne-Dejean N.; Sentef M. A.; Rubio A. Ultrafast modification of Hubbard U in a strongly correlated material: ab initio high-harmonic generation in NiO. Phys. Rev. Lett. 2018, 121, 097402 10.1103/PhysRevLett.121.097402. 30230880

[ref12] Uchida K.; Mattoni G.; Yonezawa S.; Nakamura F.; Maeno Y.; Tanaka K. High-order harmonic generation and its unconventional scaling law in the Mott-insulating Ca_2_RuO_4_. Phys. Rev. Lett. 2022, 128, 127401 10.1103/PhysRevLett.128.127401. 35394320

[ref13] Murakami Y.; Uchida K.; Koga A.; Tanaka K.; Werner P. Anomalous temperature dependence of high-harmonic generation in Mott insulators. Phys. Rev. Lett. 2022, 129, 157401 10.1103/PhysRevLett.129.157401. 36269969

[ref14] Bionta M. R.; Haddad E.; Leblanc A.; Gruson V.; Lassonde P.; Ibrahim H.; Chaillou J.; Émond N.; Otto M. R.; Jiménez-Galán A.; et al. Tracking ultrafast solid-state dynamics using high harmonic spectroscopy. Phys. Rev. Res. 2021, 3, 023250 10.1103/PhysRevResearch.3.023250.

[ref15] Neufeld O.; Zhang J.; De Giovannini U.; Hübener H.; Rubio A. Probing phonon dynamics with multidimensional high harmonic carrier-envelope-phase spectroscopy. Proc. Natl. Acad. Sci. U. S. A. 2022, 119, e2204219119 10.1073/pnas.2204219119. 35704757 PMC9231615

[ref16] Uzan A. J.; Orenstein G.; Jiménez-Galán Á.; McDonald C.; Silva R. E.; Bruner B. D.; Klimkin N. D.; Blanchet V.; Arusi-Parpar T.; Krüger M.; et al. Attosecond spectral singularities in solid-state high-harmonic generation. Nat. Photonics 2020, 14, 183–187. 10.1038/s41566-019-0574-4.

[ref17] Freudenstein J.; Borsch M.; Meierhofer M.; Afanasiev D.; Schmid C. P.; Sandner F.; Liebich M.; Girnghuber A.; Knorr M.; Kira M.; et al. Attosecond clocking of correlations between Bloch electrons. Nature 2022, 610, 290–295. 10.1038/s41586-022-05190-2. 36224421

[ref18] Bauer D.; Hansen K. K. High-harmonic generation in solids with and without topological edge states. Phys. Rev. Lett. 2018, 120, 177401 10.1103/PhysRevLett.120.177401. 29756832

[ref19] Silva R.; Jiménez-Galán Á.; Amorim B.; Smirnova O.; Ivanov M. Topological strong-field physics on sub-laser-cycle timescale. Nat. Photonics 2019, 13, 849–854. 10.1038/s41566-019-0516-1.

[ref20] Baykusheva D.; Chacón A.; Lu J.; Bailey T. P.; Sobota J. A.; Soifer H.; Kirchmann P. S.; Rotundu C.; Uher C.; Heinz T. F.; et al. All-optical probe of three-dimensional topological insulators based on high-harmonic generation by circularly polarized laser fields. Nano Lett. 2021, 21, 8970–8978. 10.1021/acs.nanolett.1c02145. 34676752

[ref21] Bai Y.; Fei F.; Wang S.; Li N.; Li X.; Song F.; Li R.; Xu Z.; Liu P. High-harmonic generation from topological surface states. Nat. Phys. 2021, 17, 311–315. 10.1038/s41567-020-01052-8.

[ref22] Heide C.; Kobayashi Y.; Baykusheva D. R.; Jain D.; Sobota J. A.; Hashimoto M.; Kirchmann P. S.; Oh S.; Heinz T. F.; Reis D. A.; et al. Probing topological phase transitions using high-harmonic generation. Nat. Photonics 2022, 16, 620–624. 10.1038/s41566-022-01050-7.

[ref23] Lakhotia H.; Kim H. Y.; Zhan M.; Hu S.; Meng S.; Goulielmakis E. Laser picoscopy of valence electrons in solids. Nature 2020, 583, 55–59. 10.1038/s41586-020-2429-z. 32612227

[ref24] Heinrich T.; Taucer M.; Kfir O.; Corkum P. B.; Staudte A.; Ropers C.; Sivis M. Chiral high-harmonic generation and spectroscopy on solid surfaces using polarization-tailored strong fields. Nat. Commun. 2021, 12, 3723 10.1038/s41467-021-23999-9. 34140484 PMC8211651

[ref25] Higuchi T.; Heide C.; Ullmann K.; Weber H. B.; Hommelhoff P. Light-field-driven currents in graphene. Nature 2017, 550, 224–228. 10.1038/nature23900. 28953882

[ref26] Heide C.; Higuchi T.; Weber H. B.; Hommelhoff P. Coherent electron trajectory control in graphene. Phys. Rev. Lett. 2018, 121, 207401 10.1103/PhysRevLett.121.207401. 30500256

[ref27] Neufeld O.; Tancogne-Dejean N.; De Giovannini U.; Hübener H.; Rubio A. Light-driven extremely nonlinear bulk photogalvanic currents. Phys. Rev. Lett. 2021, 127, 126601 10.1103/PhysRevLett.127.126601. 34597089

[ref28] Schaibley J. R.; Yu H.; Clark G.; Rivera P.; Ross J. S.; Seyler K. L.; Yao W.; Xu X. Valleytronics in 2D materials. Nat. Rev. Mater. 2016, 1, 16055 10.1038/natrevmats.2016.55.

[ref29] Ye Z.; Sun D.; Heinz T. F. Optical manipulation of valley pseudospin. Nat. Phys. 2017, 13, 26–29. 10.1038/nphys3891.

[ref30] Geondzhian A.; Rubio A.; Altarelli M. Valley selectivity of soft x-ray excitations of core electrons in two-dimensional transition metal dichalcogenides. Phys. Rev. B 2022, 106, 115433 10.1103/PhysRevB.106.115433.

[ref31] Avetissian H. K.; Mkrtchian G. F.; Batrakov K. G.; Maksimenko S. A.; Hoffmann A. Nonlinear theory of graphene interaction with strong laser radiation beyond the Dirac cone approximation: Coherent control of quantum states in nano-optics. Phys. Rev. B 2013, 88, 245411 10.1103/PhysRevB.88.245411.

[ref32] Langer F.; Schmid C. P.; Schlauderer S.; Gmitra M.; Fabian J.; Nagler P.; Schüller C.; Korn T.; Hawkins P. G.; Steiner J. T.; et al. Lightwave valleytronics in a monolayer of tungsten diselenide. Nature 2018, 557, 76–80. 10.1038/s41586-018-0013-6. 29720633 PMC6205603

[ref33] Jiménez-Galán A.; Silva R. E. F.; Smirnova O.; Ivanov M. Lightwave control of topological properties in 2D materials for sub-cycle and non-resonant valley manipulation. Nat. Photonics 2020, 14, 728–732. 10.1038/s41566-020-00717-3.

[ref34] Mrudul M. S.; Jiménez-Galán Á.; Ivanov M.; Dixit G. Light-induced valleytronics in pristine graphene. Optica 2021, 8, 422–427. 10.1364/OPTICA.418152.

[ref35] Sharma S.; Elliott P.; Shallcross S. Valley control by linearly polarized laser pulses: example of WSe2. Optica 2022, 9, 947–952. 10.1364/OPTICA.458991.

[ref36] Silva R. E. F.; Ivanov M.; Jiménez-Galán Á. All-optical valley switch and clock of electronic dephasing. Opt. Express 2022, 30, 30347–30355. 10.1364/OE.460291. 36242140

[ref37] Rana N.; Dixit G. All-optical ultrafast valley switching in two-dimensional materials. Phys. Rev. Appl. 2023, 19, 034056 10.1103/PhysRevApplied.19.034056.

[ref38] Sharma S.; Elliott P.; Shallcross S. THz induced giant spin and valley currents. Sci. Adv. 2023, 9, eadf3673 10.1126/sciadv.adf3673. 36921048 PMC10017034

[ref39] Vampa G.; Hammond T.; Thiré N.; Schmidt B.; Légaré F.; McDonald C.; Brabec T.; Klug D.; Corkum P. All-optical reconstruction of crystal band structure. Physical review letters 2015, 115, 193603 10.1103/PhysRevLett.115.193603. 26588381

[ref40] Lanin A. A.; Stepanov E. A.; Fedotov A. B.; Zheltikov A. M. Mapping the electron band structure by intraband high-harmonic generation in solids. Optica 2017, 4, 516–519. 10.1364/OPTICA.4.000516.

[ref41] Luu T. T.; Wörner H. J. Measurement of the Berry curvature of solids using high-harmonic spectroscopy. Nat. Commun. 2018, 9, 916 10.1038/s41467-018-03397-4. 29500349 PMC5834542

[ref42] Lv Y.-Y.; Xu J.; Han S.; Zhang C.; Han Y.; Zhou J.; Yao S.-H.; Liu X.-P.; Lu M.-H.; Weng H.; et al. High-harmonic generation in Weyl semimetal β-WP2 crystals. Nat. Commun. 2021, 12, 6437 10.1038/s41467-021-26766-y. 34750384 PMC8575912

[ref43] Zhou S.; Bao C.; Fan B.; Zhou H.; Gao Q.; Zhong H.; Lin T.; Liu H.; Yu P.; Tang P.; et al. Pseudospin-selective Floquet band engineering in black phosphorus. Nature 2023, 614, 75–80. 10.1038/s41586-022-05610-3. 36725995

[ref44] Hübener H.; De Giovannini U.; Schäfer C.; Andberger J.; Ruggenthaler M.; Faist J.; Rubio A. Engineering quantum materials with chiral optical cavities. Nature materials 2021, 20, 438–442. 10.1038/s41563-020-00801-7. 33168980

[ref45] Wang Y.; Steinberg H.; Jarillo-Herrero P.; Gedik N. Observation of Floquet-Bloch states on the surface of a topological insulator. Science 2013, 342, 453–457. 10.1126/science.1239834. 24159040

[ref46] Uzan-Narovlansky A. J.; Jiménéz-Galán A.; Orenstein G.; Silva R. E. F.; Arusi-Parpar T.; Shames S.; Bruner B. D.; Yan B.; Smirnova O.; Ivanov M.; et al. Observation of light-driven band structure via multiband high-harmonic spectroscopy. Nat. Photonics 2022, 16, 428–432. 10.1038/s41566-022-01010-1.

[ref47] Neufeld O.; Mao W.; Hübener H.; Tancogne-Dejean N.; Sato S. A.; De Giovannini U.; Rubio A. Time- and angle-resolved photoelectron spectroscopy of strong-field light-dressed solids: prevalence of the adiabatic band picture. Phys. Rev. Res. 2022, 4, 033101 10.1103/PhysRevResearch.4.033101.

[ref48] Ikeda T. N.; Tanaka S.; Kayanuma Y. Floquet-Landau-Zener interferometry: usefulness of the Floquet theory in pulse-laser-driven systems. Phys. Rev. Res. 2022, 4, 033075 10.1103/PhysRevResearch.4.033075.

[ref49] Soifer H.; Gauthier A.; Kemper A. F.; Rotundu C. R.; Yang S.-L.; Xiong H.; Lu D.; Hashimoto M.; Kirchmann P. S.; Sobota J. A.; et al. Band-resolved imaging of photocurrent in a topological insulator. Phys. Rev. Lett. 2019, 122, 167401 10.1103/PhysRevLett.122.167401. 31075004

[ref50] Beaulieu S.; Schusser J.; Dong S.; Schüler M.; Pincelli T.; Dendzik M.; Maklar J.; Neef A.; Ebert H.; Hricovini K.; et al. Revealing hidden orbital pseudospin texture with time-reversal dichroism in photoelectron angular distributions. Phys. Rev. Lett. 2020, 125, 216404 10.1103/PhysRevLett.125.216404. 33274965

[ref51] Graf M.; Vogl P. Electromagnetic fields and dielectric response in empirical tight-binding theory. Phys. Rev. B 1995, 51, 4940 10.1103/PhysRevB.51.4940. 9979365

[ref52] Moos D.; Jürß C.; Bauer D. Intense-laser-driven electron dynamics and high-order harmonic generation in solids including topological effects. Phys. Rev. A 2020, 102, 053112 10.1103/PhysRevA.102.053112.

[ref53] Holthaus M. Floquet engineering with quasienergy bands of periodically driven optical lattices. Journal of Physics B: Atomic, Molecular and Optical Physics 2016, 49, 013001 10.1088/0953-4075/49/1/013001.

[ref54] Oka T.; Kitamura S. Floquet engineering of quantum materials. Annual Review of Condensed Matter Physics 2019, 10, 387–408. 10.1146/annurev-conmatphys-031218-013423.

[ref55] Bao C.; Tang P.; Sun D.; Zhou S. Light-induced emergent phenomena in 2D materials and topological materials. Nature Reviews Physics 2022, 4, 33–48. 10.1038/s42254-021-00388-1.

[ref56] Rudner M. S.; Lindner N. H. Band structure engineering and non-equilibrium dynamics in Floquet topological insulators. Nature reviews physics 2020, 2, 229–244. 10.1038/s42254-020-0170-z.

[ref57] Higuchi T.; Stockman M. I.; Hommelhoff P. Strong-field perspective on high-harmonic radiation from bulk solids. Phys. Rev. Lett. 2014, 113, 213901 10.1103/PhysRevLett.113.213901. 25479494

[ref58] Ikeda T. N.; Chinzei K.; Tsunetsugu H. Floquet-theoretical formulation and analysis of high-order harmonic generation in solids. Phys. Rev. A 2018, 98, 063426 10.1103/PhysRevA.98.063426.

[ref59] Jauho A. P.; Johnsen K. Dynamical Franz-Keldysh Effect. Phys. Rev. Lett. 1996, 76, 4576–4579. 10.1103/PhysRevLett.76.4576. 10061326

[ref60] Otobe T.; Shinohara Y.; Sato S. A.; Yabana K. Femtosecond time-resolved dynamical Franz-Keldysh effect. Phys. Rev. B 2016, 93, 045124 10.1103/PhysRevB.93.045124.

[ref61] Lucchini M.; Sato S. A.; Ludwig A.; Herrmann J.; Volkov M.; Kasmi L.; Shinohara Y.; Yabana K.; Gallmann L.; Keller U. Attosecond dynamical Franz-Keldysh effect in polycrystalline diamond. Science 2016, 353, 916–919. 10.1126/science.aag1268. 27563093

[ref62] Neufeld O.; Hübener H.; Jotzu G.; De Giovannini U.; Rubio A. Band nonlinearity-enabled manipulation of Dirac nodes, Weyl cones, and valleytronics with intense linearly polarized light. Nano Lett. 2023, 23, 7568–7575. 10.1021/acs.nanolett.3c02139. 37578460 PMC10450813

[ref63] Neufeld O.; Cohen O. Background-free measurement of ring currents by symmetry-breaking high-harmonic spectroscopy. Phys. Rev. Lett. 2019, 123, 103202 10.1103/PhysRevLett.123.103202. 31573280

[ref64] Alon O. E.; Averbukh V.; Moiseyev N. Selection rules for the high harmonic generation spectra. Physical review letters 1998, 80, 3743 10.1103/PhysRevLett.80.3743. 11102225

[ref65] Neufeld O.; Podolsky D.; Cohen O. Floquet group theory and its application to selection rules in harmonic generation. Nat. Commun. 2019, 10, 405 10.1038/s41467-018-07935-y. 30679423 PMC6345759

[ref66] Sengupta P.; Pavlidis D.; Shi J. Optically adjustable valley Hall current in single-layer transition metal dichalcogenides. J. Appl. Phys. 2018, 123, 054301 10.1063/1.5004442.

[ref67] Cheng J.; Huang D.; Jiang T.; Shan Y.; Li Y.; Wu S.; Liu W.-T. Chiral selection rules for multi-photon processes in two-dimensional honeycomb materials. Optics letters 2019, 44, 2141–2144. 10.1364/OL.44.002141. 31042168

[ref68] Castro A.; Appel H.; Oliveira M.; Rozzi C. A.; Andrade X.; Lorenzen F.; Marques M. A. L.; Gross E. K. U.; Rubio A. Octopus: a tool for the application of time-dependent density functional theory. physica status solidi (b) 2006, 243, 2465–2488. 10.1002/pssb.200642067.

[ref69] Andrade X.; Strubbe D.; De Giovannini U.; Larsen A. H.; Oliveira M. J. T.; Alberdi-Rodriguez J.; Varas A.; Theophilou I.; Helbig N.; Verstraete M. J.; et al. Real-space grids and the Octopus code as tools for the development of new simulation approaches for electronic systems. Phys. Chem. Chem. Phys. 2015, 17, 31371–31396. 10.1039/C5CP00351B. 25721500

[ref70] Tancogne-Dejean N.; Oliveira M. J. T.; Andrade X.; Appel H.; Borca C. H.; Le Breton G.; Buchholz F.; Castro A.; Corni S.; Correa A. A.; et al. Octopus, a computational framework for exploring light-driven phenomena and quantum dynamics in extended and finite systems. J. Chem. Phys. 2020, 152, 124119 10.1063/1.5142502. 32241132

[ref71] Hartwigsen C.; Gœdecker S.; Hutter J. Relativistic separable dual-space Gaussian pseudopotentials from H to Rn. Phys. Rev. B 1998, 58, 3641 10.1103/PhysRevB.58.3641. 9986014

[ref72] Dong S.; Beaulieu S.; Selig M.; Rosenzweig P.; Christiansen D.; Pincelli T.; Dendzik M.; Ziegler J. D.; Maklar J.; Xian R. P. Observation of ultrafast interfacial Meitner-Auger energy transfer in a van der Waals heterostructure. arXiv 2021, 10.48550/arXiv.2108.06803. PMC10439896 37598179

