# Supplementary material for: Mapping Light-Dressed Floquet Bands by Highly Nonlinear Optical Excitations and Valley Polarization
Source: J Phys Chem Lett. 2023 Dec 8;14(50):11298–304. doi: 10.1021/acs.jpclett.3c02936 (PMC10749462; doi:10.1021/acs.jpclett.3c02936)
Supplement: Supplementary file 1 — jz3c02936_si_001.pdf [file jz3c02936_si_001.pdf]

Supplementary Material for "Mapping Light-Dressed Floquet Bands by Highly Nonlinear Optical Excitations and Valley Polarization"

Anna Galler,[*,†] Angel Rubio,[†,‡] and Ofer Neufeld[†]

†*Max Planck Institute for the Structure and Dynamics of Matter, Center for Free Electron Laser Science, 22761 Hamburg, Germany*

‡*Center for Computational Quantum Physics, Flatiron Institute, 162 5th Avenue, New York, NY 10010, USA*

E-mail: anna.galler@mpsd.mpg.de

In this supplementary material, we present technical details concerning the calculations presented in the main text, as well as additional results underlining our main findings.

Model calculations

As described in the main text, we employed a real-space 2D model for a honeycomb system with different A/B sublattice sites. The potential on each site is given by a Gaussian of the form:

$$V_{A,B}(\mathbf{r}) = -v_{0,A,B} e^{-\mathbf{r}^2/\sigma_{A,B}^2} \tag{1}$$

where $\mathbf{r}$ is the electronic coordinate in 2D, $v_{0,A} = 40$ eV, $v_{0,B} = 1.08 v_{0,A}$, $\sigma_A = 1.5$ Bohr, and $\sigma_B = 1.08\sigma_A$, such that the sublattice sites differ by 8%. The lattice vectors employed were of length 2π Bohr, of the form $\mathbf{a_1} = 2\pi\hat{\mathbf{x}}$, $\mathbf{a_2} = 2\pi(-\hat{\mathbf{x}}/2 + \sqrt(3)\hat{\mathbf{y}}/2)$. Each site contributes one electron to the unit cell, such that there are two electrons in each cell that populate the same band (the first valence band), with spin-orbit interactions and electron-electron interactions fully neglected. The field-free Hamiltonian of the model in atomic units in the real-space representation reads:

$$h_0(\mathbf{r}) = \sum_{n,m} V_A(\mathbf{r} - n\mathbf{a_1} - m\mathbf{a_2}) + \sum_{n,m} V_B(\mathbf{r} - n\mathbf{a_1} - m\mathbf{a_2} - \frac{\mathbf{a_1}}{3} + \frac{\mathbf{a_2}}{3}) - \frac{1}{2}\nabla^2 \tag{2}$$

where n and m are integers. We employed periodic boundary conditions along the lattice vectors and diagonalized the Hamiltonian to obtain the ground state Bloch states with a real-space grid spacing of 0.28 Bohr, and a k-space discretization using a Γ-centered 60×60 k-grid.

For the time-dependent calculations, we solved the time-dependent Schrödinger equation (TDSE) while propagating the Bloch state that is initially associated with the valence band

in the real-space grid representation. The TDSE in atomic units is given by:

$$i\partial_t |\psi_k(t)\rangle = \left[\sum_{n,m} V_A(\mathbf{r} - n\mathbf{a_1} - m\mathbf{a_2})\right] |\psi_k(t)\rangle + \\ + \left[\sum_{n,m} V_B(\mathbf{r} - n\mathbf{a_1} - m\mathbf{a_2} - \frac{\mathbf{a_1}}{3} + \frac{\mathbf{a_2}}{3}) + \frac{1}{2}(-i\nabla - \frac{1}{c}\mathbf{A}(t))^2\right] |\psi_k(t)\rangle \quad (3)$$

where we employ the velocity gauge and dipole approximation for the laser-matter interaction, and $\mathbf{A}(t)$ is the vector potential of the laser electric field such that $-\partial_t\mathbf{A}(t) = c\mathbf{E}(t)$, with c is the speed of light. The initial state is taken as the ground state and propagation is performed with a Lanczos expansion method with a time-step of 0.2 a.u. The laser vector potential employed was taken as:

$$\mathbf{A}(t) = f(t)\frac{cE_0}{\omega\sqrt{1+\epsilon^2}}\hat{R(\theta)} \cdot (\cos(\omega t)\hat{\mathbf{x}} + \epsilon\sin(\omega t)\hat{\mathbf{y}}) \quad (4)$$

where E_0 is the electric field amplitude, ω is the driving frequency, ϵ is the field's ellipticity, and $\hat{R(\theta)}$ is a rotation matrix in the xy-plane operating on the polarization vector. Overall, this vector potential describes an elliptically-polarized laser pulse with an ellptical major axis oriented θ degrees above the x-axis. $f(t)$ in Eq.(4) is a normalized temporal envelope function taken as a super-sine form[1] (see illustration in Fig. S1):

$$f(t) = \sin\left(\pi\frac{t}{T_p}\right)^{\left(\frac{|\pi(\frac{t}{T_p}-\frac{1}{2})|}{\sigma}\right)}, \quad (5)$$

where $\sigma = 0.75$, T_p is the duration of the laser pulse which was taken to be $T_p = 10T$, where T is a single cycle of the fundamental carrier frequency (the overall full-width-half-max of the pulse is 5T). The shape of an exemplary linearly-polarized laser pulse is shown in Fig. S1.

Projections of the occupations over the CB after the pulse ends were calculated by pro-

jecting the time-dependent Bloch state over the ground state band:

$$g_{CB}(\mathbf{k}) = |\langle\psi_{CB,\mathbf{k}}(t=0)|\psi_{\mathbf{k}}(t_{end})\rangle|^2 \tag{6}$$

Note that in this approach each k-point is numerically independent such that the equations of motion for the different Bloch states are propagated separately. Moreover, due to the dipole approximation it is assumed that optical transitions can only occur while preserving the electron's momentum, i.e. vertically (as discussed in the main text). All of the numerical calculations were performed with `Octopus` code.[2–4]

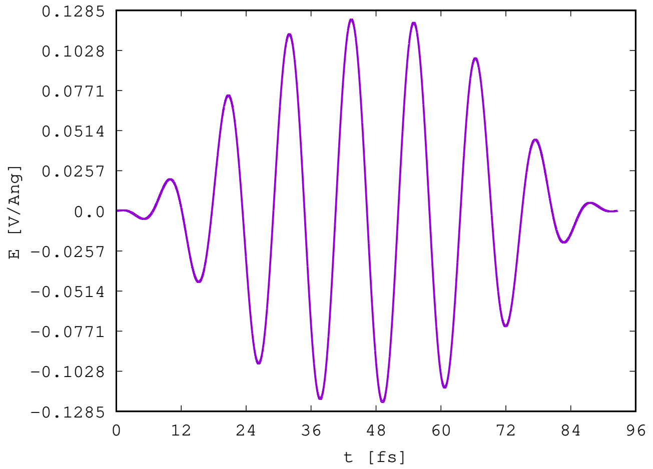

Figure S1: Electric field $E(t)$ of a linearly polarized pump pulse with $\omega = 0.35\,\text{eV}$, maximum field intensity $0.2\,\text{TW/cm}^2$ and a super-sine envelope function as specified in Eq. 5

TDDFT calculations

Time-dependent density functional theory (TDDFT) calculations were performed using the real-space grid-based code `Octopus`.[2–4] The Kohn Sham (KS) equations were discretized on a 3D Cartesian grid in the primitive unit cell of monolayer hexagonal boron nitride (hBN), with the lattice parameter $a = 2.52$ Å taken as the experimental value. A vacuum spacing of 40 Bohr was included in the simulations above and below the monolayer. Calculations were

performed using the local density approximation (LDA), and spin degrees of freedom and spin-orbit coupling were neglected. The frozen core approximation was used for inner core bands which were treated with norm-conserving pseudopotentials.[5] The KS equations were solved self-consistently with an energy tolerance of $< 10^{-7}$ Hartree, and the grid spacing was converged to 0.37 Bohr. We employed a Γ-centred k-grid with 72×72 k-points. Fig. S2 presents the obtained ground state band structure in hBN.

For the TDDFT calculations, the time-dependent KS equations were solved within the adiabatic approximation, represented in real-space and in the velocity gauge, given in atomic units by

$$i\partial_t \left|\psi_{n,k}^{KS}(t)\right\rangle = \left[\frac{1}{2}\left(-i\nabla - \frac{1}{c}\mathbf{A}(t)\right)^2 + v_{KS}(\mathbf{r},t)\right]\left|\psi_{n,k}^{KS}(t)\right\rangle, \tag{7}$$

where $\left|\psi_{n,k}^{KS}(t)\right\rangle$ are the KS single-particle wave functions of band n, $\mathbf{A}(t)$ is the vector potential of the laser field (with the same form as used for the model TDSE calculations described above), and $v_{KS}(\mathbf{r},t)$ the time-dependent KS potential given by

$$v_{KS}(\mathbf{r},t) = -\sum_I \frac{Z_I}{|\mathbf{R}_I - \mathbf{r}|} + \int d^3r' \frac{n(\mathbf{r}',t)}{|\mathbf{r}-\mathbf{r}'|} + v_{XC}[n(\mathbf{r},t)], \tag{8}$$

where Z_I is the charge of the Ith nuclei and $\mathbf{R}_I$ is its coordinate, v_{XC} is the XC potential that is a functional of the time-dependent electron density $n(\mathbf{r},t) = \sum_{n,k} | \left|\psi_{n,k}^{KS}(t)\right\rangle |^2$. Practically, the Coulomb interaction with the nuclei (first term in Eq.(8)) was replaced by the non-local pseudopotential that also incorporates the core electrons contributions. The KS wave functions were propagated with a time-step of $\Delta t = 0.2$ a.u. The initial state was taken to be the system's ground state. The propagator was represented by a Lanczos expansion. Absorbing boundaries with a width of 12 Bohr were employed along the direction of the non-periodic z-axis.

Band and k-point occupations were calculated by projecting the time-dependent KS states on the field-free states at $t = 0$, just as discussed above for the model case, but with multiple band contributions taken into account.

As a quantitative measure for the valley asymmetry we define $P = (n_K - n_{K'})/(n_K + n_{K'})$, where n_K ($n_{K'}$) are the electron occupations obtained by integrating the conduction band population in the half of the reciprocal unit cell containing K (K').

Tight-binding Hamiltonian and Floquet analysis

We employed a two-band tight-binding Hamiltonian with up to 5th order NN contributions to describe the Gaussian honeycomb model, as well as hBN (following ref.[6]). In the basis of creation/annihilation operators on the A/B sublattice sites, the TB Hamiltonian reads:

$$H(\mathbf{k}) = \begin{pmatrix} t_2 f_2(\mathbf{k}) + t_5 f_5(\mathbf{k}) & t_1 f_1(\mathbf{k}) + t_3 f_3(\mathbf{k}) + t_5 f_5(\mathbf{k}) \\ t_1 f_1^*(\mathbf{k}) + t_3 f_3^*(\mathbf{k}) + t_5 f_5^*(\mathbf{k}) & t_2 f_2(\mathbf{k}) + t_5 f_5(\mathbf{k}) \end{pmatrix} + \\ + (3t_2 - 6t_5 + \Delta/2)\sigma_0 + \Delta\sigma_z/2 \tag{9}$$

where σ_0 is the 2-by-2 identity matrix, t_i are the hopping amplitudes to the i'th NN site (set as 1.5289, 0.1416, 0.0777, 0.0177, 0.0124 eV for t_1, t_2, t_3, t_4, and t_5, respectively for the Gaussian honeycomb model, and as -2.1429, 0.3376, -0.0630, 0.1825, 0.0928 eV, respectively, for hBN), and $f_i(\mathbf{k})$ are structure factors obtained by Fourier transforming the real-space Hamiltonian with the form:

$$f_m(\mathbf{k}) = \sum_j e^{i\mathbf{k}\cdot\mathbf{d}_{m,j}} \tag{10}$$

where the sum runs over existing j indices that describe various NN vectors for order m hoppings, and $\mathbf{d}_{m,j}$ are the NN hopping vectors of order m. The hopping vectors are given

by:

$$d_{1,1} = (0, 1/\sqrt{3}), d_{1,2} = (-1, -1/\sqrt{3})/2, d_{1,3} = (1, -1/\sqrt{3})/2$$

$$d_{2,1} = (-1, -\sqrt{3})/2, d_{2,2} = (1, \sqrt{3})/2, d_{2,3} = (-1, 0), d_{2,4} = (1, 0)$$

$$d_{2,5} = (-1, \sqrt{3})/2, d_{2,6} = (1, -\sqrt{3})/2$$

$$d_{3,1} = (1, 1/\sqrt{3}), d_{3,2} = (-1, -1/\sqrt{3}), d_{3,3} = (0, -2/\sqrt{3})$$

$$d_{4,1} = (-1, -2/\sqrt{3}), d_{4,2} = (1/2, 5/(2\sqrt{3})), d_{4,3} = (-3/2, -1/(2\sqrt{3}))$$

$$d_{4,4} = (3/2, -1/(2\sqrt{3})), d_{4,5} = (-1/2, 5/(2\sqrt{3})), d_{4,6} = (1, -2/\sqrt{3})$$

$$d_{5,1} = (-3/2, -\sqrt{3}/2), d_{5,2} = (3/2, \sqrt{3}/2), d_{5,3} = (3/2, -\sqrt{3}/2)$$

$$d_{5,4} = (-3/2, \sqrt{3}/2), d_{5,5} = (0, \sqrt{3}), d_{5,6} = (0, -\sqrt{3})$$

where all NN vectors are given in units of the lattice parameter (2π Bohr for the Gaussian honeycomb model, and $a = 2.52\,\text{Å}$ for hBN), and in the basis of Cartesian components in 2D, i.e. $(\hat{x}, \hat{y})$. The first term in Eq. (9) represents all hopping processes, where 1st, 3rd, and 4th NN transitions are A-B inter-sublattice hoppings that couple to $\sigma_{(x/y)}$ Pauli matrices, while 2nd and 5th NN transitions are A-A and B-B intra-sublattice hoppings that couple to σ_0. The second term in Eq. (9) shifts the Fermi level to the top of the valence band for convenience purposes, and is otherwise physically irrelevant. The third term opens a gap of Δ at the K/K' points in the BZ. Note that the choice of hopping amplitudes was set such that the TB model bands reproduce the first valence and conduction bands from the Gaussian honeycomb model, or hBN, by a least-squares fitting procedure. For hBN the least-squares fitting procedure was only employed in the region around the K/K' valleys to avoid fitting the inverted band structure around Γ.

The Floquet analysis with the TB Hamiltonians is performed as discussed in the main text, where the Fourier decomposition integrals (Eq.(1) in the main text) are performed numerically for each k-point via Simpson integration with 200 points per cycle, and the maximal allowed photon channel was 10ω. All parameters were tested for convergence. Fig.

S3 presents and examplay Floquet band structure obtained for the model system.

Additional results

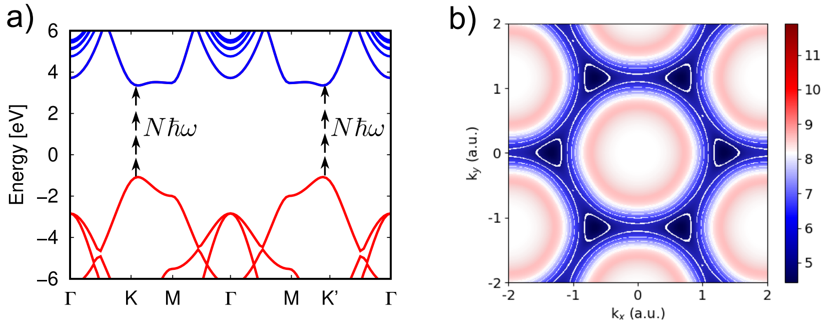

Figure S2: (a) Equilibrium band structure of monolayer hBN, computed within LDA, shown along high-symmetry lines (red and blue denoting occupied and unoccupied states, respectively). Multiple photons with sub-band-gap frequency ω are needed to excite electrons from the valence to the conduction bands. (b) Equilibrium band gap of hBN in the $k_x k_y$ plane. The white lines indicate direct gap energies resonant with multiples of the driving frequency $\omega = 0.7\,\text{eV}$.

Here, we present additional results supporting the findings presented in the main text. A comparison between the equilibrium and the Floquet driven band structure along high symmetry lines in the BZ is shown in Fig. S3 for exemplary conditions. The shape of the depicted Floquet driven valence and conduction bands are for a linearly polarized laser in the x direction, in accordance with Fig. 2 in the main text.

In Fig. S4, we explicitly show that the undressed field-free states do not satisfactorily describe the induced charge excitation patterns. Fig. S4 depicts exemplary charge excitation patterns in the model, overlaid with multi-photon resonant lines in the equilibrium (field-free) band gap. The excitation patterns do not match the resonant lines, in particular for elliptical (Fig. S4b) and linear (Fig. S4c) driving, the patterns break the expected three-fold rotational symmetry of the field-free system. This discrepancy can be mended by considering the light-dressed Floquet band structure instead, as discussed in the main text.

In Figs. (S5) and (S6), we show further results for various driving frequencies and power.

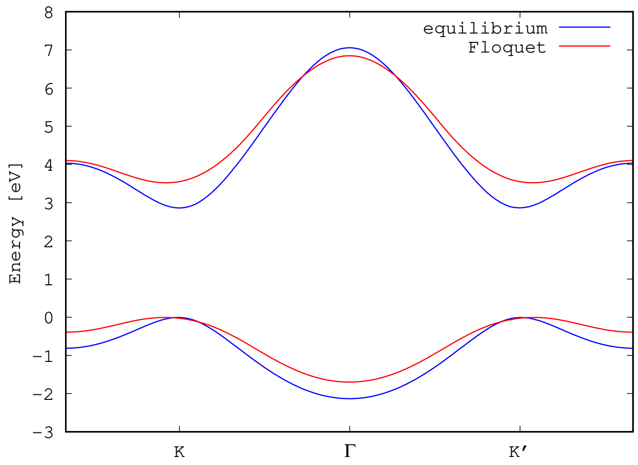

Figure S3: Comparison between the equilibrium electronic bands and the Floquet driven bands in the model along high symmetry lines. The depicted Floquet driven bands are calculated for a laser linearly polarized in x-direction with $\omega = 0.35\,\text{eV}$ and $0.2\,\text{TW/cm}^2$, similarly to Fig. 2 in the main text.

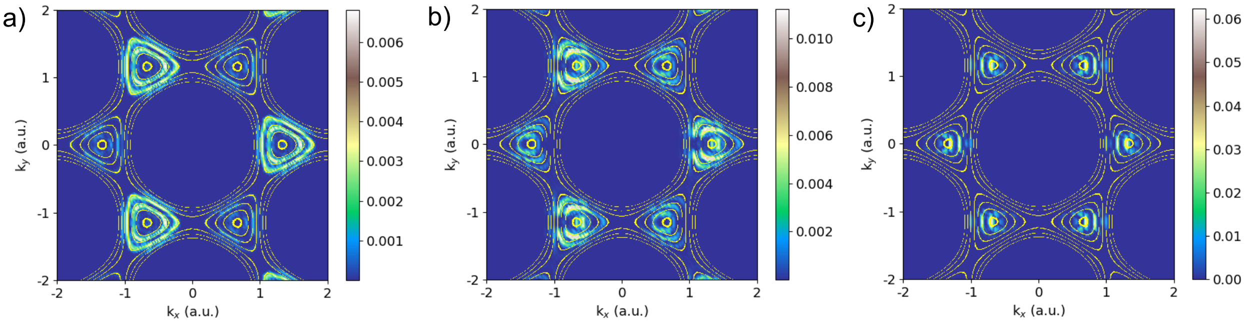

Figure S4: Charge excitation patterns in the model after an intense ($0.2\,\text{TW/cm}^2$) pump pulse with (a) circular polarization, (b) elliptical polarization with $\epsilon = 0.6$, and (c) linear polarization in x-direction. In all panels, the driving frequency is $0.35\,\text{eV}$, the overlaid yellow lines represent multi-photon resonant energies in the equilibrium band structure. In all panels, the charge excitation patterns do not satisfactorily match the resonance lines, for (b) elliptical and (c) linear driving the patterns break the three-fold rotation symmetry of the equilibrium band gap.

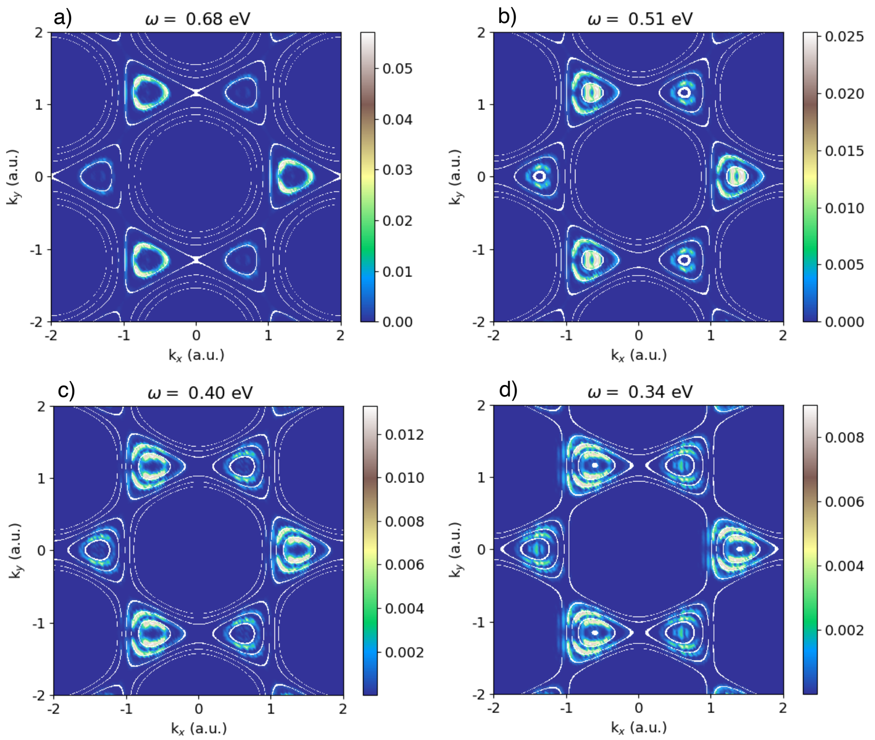

Figure S5: Conduction band excitation patterns in the model after an intense ($0.2\,\mathrm{TW/cm^2}$) elliptically polarized ($\epsilon = 0.6$) pump pulse with frequencies (a) $0.68\,\mathrm{eV}$, (b) $0.51\,\mathrm{eV}$, (c) $0.40\,\mathrm{eV}$, and (d) $0.34\,\mathrm{eV}$. In all cases, the charge excitation patterns almost perfectly match the Floquet multi-photon resonant lines in the Brillouin zone, indicated by the white lines.

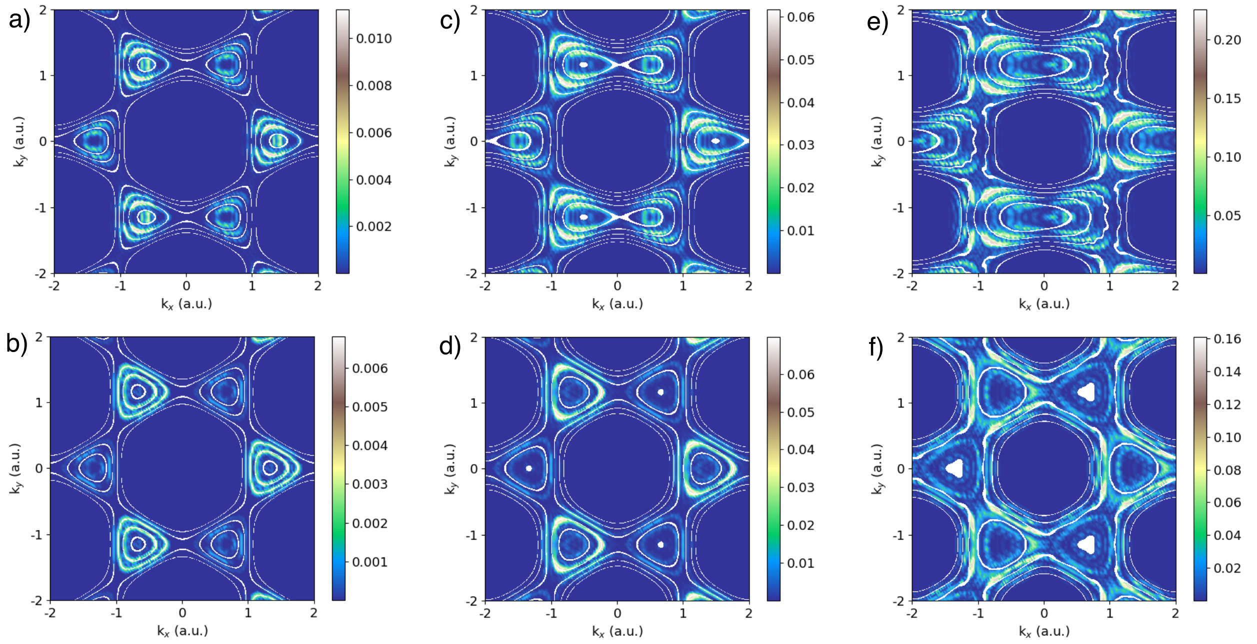

Figure S6: Conduction band charge excitation patterns in the model for three different laser driving powers: (a-b) $0.2\,\mathrm{TW/cm^2}$, (c-d) $0.4\,\mathrm{TW/cm^2}$, and (e-f) $0.7\,\mathrm{TW/cm^2}$. The panels in the first row are for an elliptically polarized pump pulse ($\epsilon = 0.6$), while the second row depicts results for circular polarization, the driving frequency is $0.35\,\mathrm{eV}$ in all panels. For circularly polarized pump pulses (second row), the valley polarization is clearly visible.

Fig. (S5) displays conduction band charge excitation patterns in the model induced by an elliptically polarized pump pulse with various driving frequencies ω. For all investigated ω, the charge patterns nicely match the Floquet multi-photon resonant lines in the BZ. Fig. (S6) depicts the charge excitation patterns in the model induced by elliptically as well as circularly polarized pump pulses with different driving powers. These additional results further support the validity of the interpretation that the induced charge patterns directly map transitions between the Floquet driven bands.

We further verified that our results do not significantly depend on the duration of the pump pulse. Fig. S7a) shows that the characteristic ring pattern appears already after a short 3-cycle elliptically-polarized pump pulse. The pattern consequently becomes more defined with increasing number of pump cycles, see Fig. S7b-c).

We next explore the dependence of the results on the TB model parameters. While the employed TB model describes very well the equilibrium electronic bands, the Bloch states are not perfectly described, and their phases can be inaccurate. These phases can be crucial for multi-photon transitions in certain regimes. In Fig. S8 we show how sensitive the Floquet bands are to the TB model—by changing the number of nearest-neighbour interactions from two (see Fig. S8a) to five (see Fig. S8b), the resonant lines substantially move, inducing potential missmatches between the Floquet resonant lines and the numerically-exact occupation patterns. Such issues can in principle be avoided by utilizing other forms for the Floquet Hamiltonian, or other forms of models. However, we would like to emphasize that the mismatches are anyways relatively small and only reflect the position of the resonant lines, while the general structure and shape of the pattern remains in very good agreement with the shape of the Floquet-TB bands (as illustrated in Fig. S8(c) that shows a denser contour plot of the Floquet k-dependent pseudo-energy gap.

In Fig. S9, we show limiting parameter regimes for our interpretation of the observed charge patterns as Floquet multi-photon resonant lines. For weak power and high frequency driving, as shown in Fig. S9a), the Floquet bands converge to the field-free bands, and all

of the interesting observed effects simply vanish, since expressions for Fermi's golden rule coincide with those for the non-driven system (the Floquet picture can be considered a generalization of the standard approach). On the other hand, in the limit of extreme driving (see Fig. S9b), the Floquet picture becomes much more complicated due to the involvement of multiple bands, and multiple band replicas, in the dynamics, which interfere. Indeed, Fig. S9b) shows that as the pump becomes very intense, the agreement between the Floquet resonant multi-photon lines and the numerical results diminishes (although remarkably, the general structure of the occupation pattern still resembles the Floquet bands, see Fig. S9b-c).

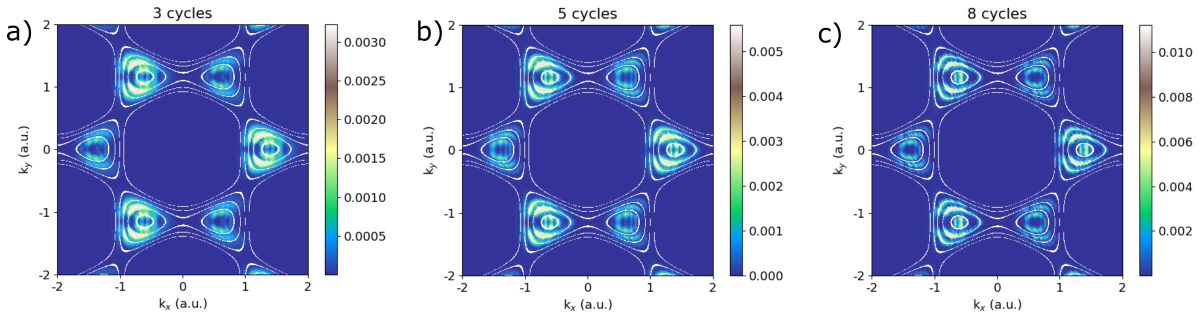

Figure S7: Conduction band excitation patterns in the model for different duration of the pump pulse: (a) 3 cycles, (b) 5 cycles, and (c) 8 cycles. In all panels, the pump pulse is elliptically polarized ($\epsilon = 0.6$) with a driving frequency 0.35 eV and driving power 0.2 TW/cm^2.

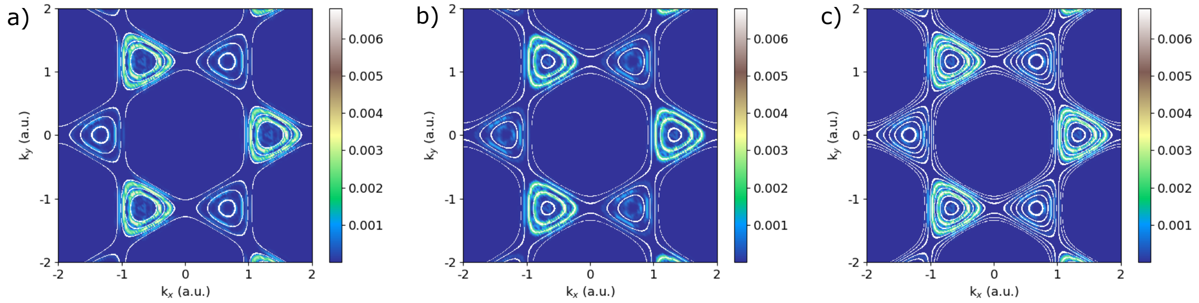

Figure S8: Dependence of the Floquet band structure on the number of nearest neighbor interactions included in the tight-binding Hamiltonian, for a circularly polarized pump pulse with 0.35 eV, 0.2 TW/cm^2 (a) two nearest-neighbor, and (b) five nearest-neighbor interactions, respectively. (c) Same as (b), but multi-photon resonant lines shown for 0.175 eV.

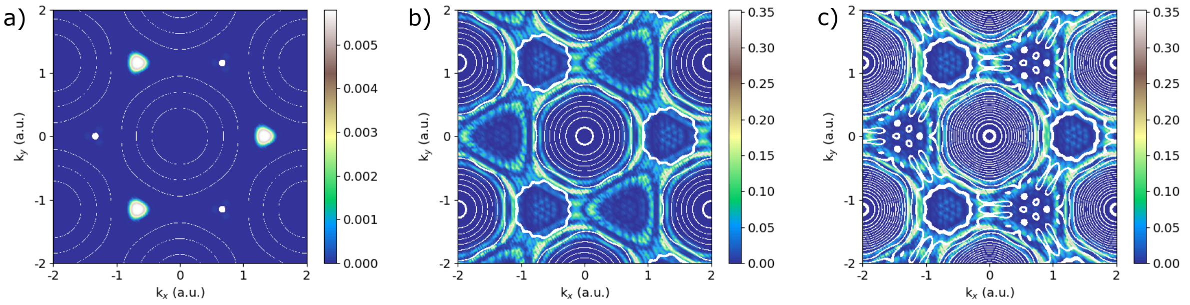

Figure S9: Limiting parameter regimes for the Floquet picture. (a) Conduction band excitation pattern in the model for circularly polarized high-frequency driving with 0.8 GW/cm^2 and 2.06 eV, where the Floquet bands converge to the field-free bands. (b) Under extreme driving with 1 TW/cm^2 and 0.35 eV, the agreement between the Floquet picture and the observed charge excitation patterns breaks down. Nevertheless, some general features can still be recognized. (c) Same as (b), but multi-photon lines shown for 0.15 eV.

References

(1) Neufeld, O.; Cohen, O. Background-Free Measurement of Ring Currents by Symmetry-Breaking High-Harmonic Spectroscopy. *Phys. Rev. Lett.* **2019**, *123*, 103202.

(2) Castro, A.; Appel, H.; Oliveira, M.; Rozzi, C. A.; Andrade, X.; Lorenzen, F.; Marques, M. A. L.; Gross, E. K. U.; Rubio, A. Octopus: a tool for the application of time-dependent density functional theory. *physica status solidi (b)* **2006**, *243*, 2465–2488.

(3) Andrade, X.; Strubbe, D.; De Giovannini, U.; Larsen, A. H.; Oliveira, M. J. T.; Alberdi-Rodriguez, J.; Varas, A.; Theophilou, I.; Helbig, N.; Verstraete, M. J. et al. Real-space grids and the Octopus code as tools for the development of new simulation approaches for electronic systems. *Phys. Chem. Chem. Phys.* **2015**, *17*, 31371–31396.

(4) Tancogne-Dejean, N.; Oliveira, M. J. T.; Andrade, X.; Appel, H.; Borca, C. H.; Le Breton, G.; Buchholz, F.; Castro, A.; Corni, S.; Correa, A. A. et al. Octopus, a computational framework for exploring light-driven phenomena and quantum dynamics in extended and finite systems. *The Journal of Chemical Physics* **2020**, *152*, 124119.

(5) Hartwigsen, C.; Gœdecker, S.; Hutter, J. Relativistic separable dual-space Gaussian pseudopotentials from H to Rn. *Physical Review B* **1998**, *58*, 3641.

(6) Wang, Y.; Huang, C.; Li, D.; Li, P.; Yu, J.; Zhang, Y.; Xu, J. Tight-binding model for electronic structure of hexagonal boron phosphide monolayer and bilayer. *Journal of Physics: Condensed Matter* **2019**, *31*, 285501.
